# Knockdown of USP7 alleviates atherosclerosis in ApoE-deficient mice by regulating EZH2 expression

**DOI:** 10.1515/biol-2022-0929

**Published:** 2024-09-19

**Authors:** Yu Zhang, Yanchun Zhang

**Affiliations:** Department of Cardiology, The Affiliated Wuxi People’s Hospital of Nanjing Medical University, Wuxi People’s Hospital, Wuxi Medical Center, Nanjing Medical University, Wuxi, Jiangsu, 214000, China; Department of Cardiology, Shuyang Hospital of Traditional Chinese Medicine, No. 28 Shanghai Road, Shuyang, Jiangsu, 223600, China

**Keywords:** atherosclerosis, ubiquitin-specific protease 7, inflammation, oxidative stress, EZH2

## Abstract

Atherosclerosis (AS) is a chronic vascular disease associated with lipid accumulation. Understanding the molecular mechanisms of AS is essential. Ubiquitin-specific protease 7 (USP7) is a deubiquitination enzyme involved in various cellular processes, including lipid metabolism. In this study, we aimed to elucidate the role of USP7 in AS progression and its underlying mechanism using ApoE-deficient mice. We found that USP7 ablation improved the morphological characteristics of AS in these mice. USP7 knockdown reduced inflammation, evidenced by decreases in inflammatory markers IL-6, TNF-α, and IL-1β by 35, 40, and 38%, respectively (*p* < 0.01). Additionally, USP7 depletion reduced oxidative stress, indicated by a 30% reduction in malondialdehyde levels and increases in superoxide dismutase and glutathione peroxidase levels by 25 and 28%, respectively (*p* < 0.01). Moreover, USP7 knockdown blocked lipid accumulation in aortic tissue cells. Mechanistically, USP7 knockdown inhibited enhancer of Zeste Homolog 2 (EZH2) expression, thereby suppressing AS progression. In conclusion, USP7 depletion alleviated AS progression in ApoE-deficient mice by targeting EZH2 expression. USP7 may serve as a therapeutic target for AS.

## 1 Introduction

Atherosclerosis (AS) is a chronic vascular disease characterized by lipid accumulation, uptake of low-density lipoprotein (LDL) by macrophages, and subsequent transformation into foam cells [1]. Its pathology includes three main features: inflammatory responses, lipid accumulation, and arterial wall fibrosis, which form the basis of coronary heart disease [2]. The cholesterol reverse transport system, which mobilizes cholesterol on high-density lipoprotein particles from extravascular tissues to the plasma, is essential in the progression of AS [3,4]. Since the exact pathology of AS is not fully understood, it is essential to elucidate its molecular mechanisms and explore therapeutic options that could promote reverse cholesterol transport and inhibit intracellular lipid accumulation.

Enhancer of Zeste Homolog 2 (EZH2) is the catalytic subunit of Polycomb Repressive Complex 2 (PRC2), responsible for catalyzing H3K27 methylation, leading to gene silencing [5,6]. EZH2 plays a significant role in epigenetic maintenance and mediates various cellular processes in certain diseases. Increased expression of EZH2 promotes AS formation in mice [6–10]. Silencing EZH2 reduces serum cholesterol levels, inhibits the inflammatory response in AS mice, promotes growth, inhibits apoptosis in OX-LDL-treated human aortic smooth muscle cells, and reduces oxidative stress in the arterial tissue of AS mice [7].

In recent years, deubiquitinating enzymes (DUBs) have received significant attention due to their impact on the progression of vascular diseases. DUBs play crucial roles in AS, abdominal aortic aneurysms, angiogenesis, and hypertension, highlighting their potential therapeutic value in vascular diseases [11]. The ubiquitin-specific protease (USP) family is the largest group of human DUBs, consisting of more than 30 members. Among these, ubiquitin-specific protease 7 (USP7) is a well-characterized deubiquitination enzyme. Recent findings have linked the regulatory role of USP7 to lipid metabolism in the liver, demonstrating its involvement in hepatic steatosis [12]. USP7 knockdown inhibits pulmonary artery smooth muscle cell proliferation caused by pulmonary hypertension [13]. Additionally, studies have shown that USP7 promotes hypoxia-induced myocardial cell damage, apoptosis, and increased secretion of inflammatory factors in cardiovascular diseases [14]. Furthermore, USP7 depletion has been associated with reduced EZH2 levels in HCT116 cancer cells [15]. However, the role and mechanism of USP7 in AS remain unclear.

In this study, we aimed to clarify the role of USP7 in the progression of AS and the underlying mechanisms. We hypothesize that USP7 plays a critical role in AS progression by regulating EZH2 expression.

## 2 Materials and methods

### 2.1 Animal models and adenovirus infection

The animal experiments were approved by the Animal Ethics Committee of the Affiliated Wuxi People’s Hospital of Nanjing Medical University. Male wild-type C57BL/6 mice and ApoE−/− C57BL/6 mice, aged 6–8 weeks, were obtained from Vitong Lihua Company (Beijing, China). After a week of acclimatization, the ApoE−/− mice were fed a Western diet containing 21% fat and 0.15% cholesterol to induce AS. In contrast, the wild-type C57BL/6 mice were fed a normal diet and served as the control group. The feeding period lasted for 12 weeks. The study included four groups, each consisting of six mice: (1) Normal, (2) AS, (3) AS + sh-negative control (NC) (non-targeting shRNA), and (4) AS + sh-USP7 (USP7 knockdown). The sample size was determined based on previous studies and a power analysis to ensure adequate statistical power for detecting significant differences between the groups.

Adenovirus plasmids for sh-NC and sh-USP7 were constructed by Heyuan Biology (Shanghai, China) following the manufacturer’s guidelines. The sh-NC adenovirus was used as a control to account for any nonspecific effects of adenovirus infection to ensure that the observed effects were specifically due to USP7 knockdown. The efficiency of USP7 knockdown was confirmed by immunoblot assays in the aortic tissues of AS mice that were resected after the experimental period.


**Ethical approval:** The research related to animal use has been complied with all the relevant national regulations and institutional policies for the care and use of animals, and has been approved by the Animal Ethics Committee of the Affiliated Wuxi People’s Hospital of Nanjing Medical University.

### 2.2 Histological analysis

The aortic tissues were resected and fixed with 5% paraformaldehyde. After fixation, the samples were embedded in paraffin, cut into slices, and counterstained with hematoxylin and eosin (H&E).

### 2.3 ELISA

The levels of IL-1β, TNF-α, and IL-6 in serum and aortic tissues were assessed using ELISA kits according to the manufacturer’s guidelines. Briefly, the aortic samples were homogenized, and the homogenates were aspirated into the wells. The following primary antibodies at corresponding dilutions were used: IL-1β (1:1,000), TNF-α (1:1,000), and IL-6 (1:1,000). Then, they were incubated with biotin-conjugated secondary antibodies and avidin-conjugated horseradish peroxidase. Next, the enzyme substrate was added for color development, and the intensity of each well was measured using a microplate reader.

### 2.4 Reactive oxygen species (ROS) detection

The levels of superoxide dismutase (SOD), glutathione peroxidase (GSH-Px), and malondialdehyde (MDA) were measured using detection kits from Nanjing Jiancheng Bio-engineering Institute (Jiangsu, China) according to the manufacturer’s guidelines. Total cholesterol (TC) and triglyceride (TG) levels were assessed using corresponding kits from Abcam (ab65390). To assess ROS levels, the samples were sectioned and stained with dihydroethidium (DHE). In an independent experiment, DHE fluorescence in five random fields was quantified using AxioVision software (Zeiss). The quantification involved measuring the positive area by setting a threshold and referencing the results to the total area.

### 2.5 TC, TG detection and Oil Red O staining

TC and TG levels were assessed using kits from Abcam (ab65390) following the manufacturer s instructions. For Oil Red O staining, aortic tissues were harvested and immediately frozen in optimal cutting temperature compound. Cryosections of the aortic tissues were then prepared and stained with Oil Red O to assess lipid accumulation. The stained sections were visualized and analyzed under a microscope.

### 2.6 Immunoblot assay

Proteins were extracted using radio immunoprecipitation assay buffer (Beyotime). The tissue samples were then collected, electrophoresed, and transferred onto polyvinylidene fluoride membranes. The membranes were blocked with 5% fat-free milk, followed by incubation with primary antibodies targeting USP7 (ab108931, 1:500; Abcam, Cambridge, UK), EZH2 (ab307646, 1:1,000; Abcam), and GAPDH (ab8245, 1:3,000; Abcam) for 2 h. Afterward, the membranes were incubated with secondary antibodies for 1 h. The blots were then analyzed using an enhanced chemiluminescence kit.

### 2.7 Statistics

Statistical analysis was performed using the GraphPad Prism 5.0 software. Data are represented as mean ± SD. Comparisons between groups were made using one-way ANOVA followed by Tukey’s *post hoc* test. *p* < 0.05 was considered statistically significant.

## 3 Results

### 3.1 Ablation of USP7 improved the AS morphological characteristics in AS mice

**Figure 1:**
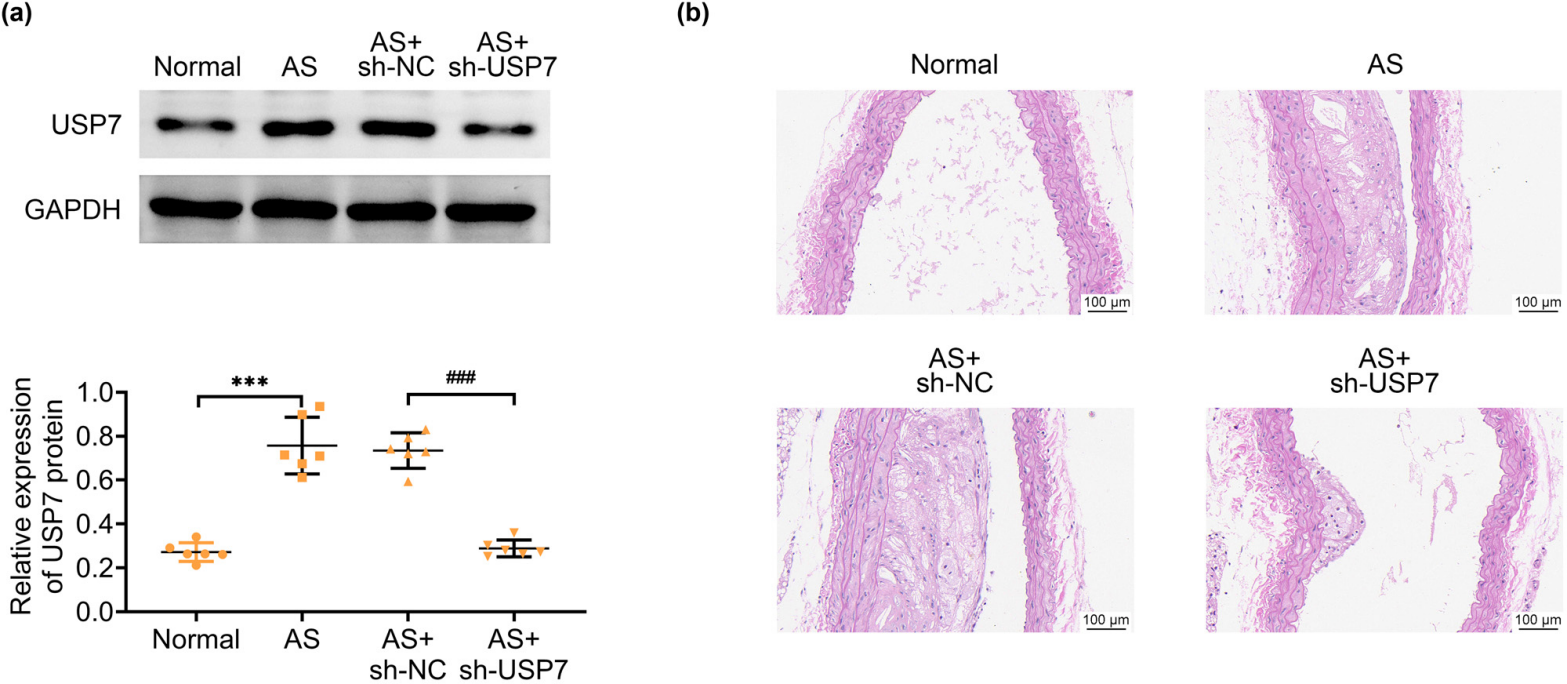
Ablation of USP7 improved the AS morphological characteristics in AS mice. (a) Immunoblot assays showed the expression of USP7 in aortic tissues of normal mice and AS (ApoE-deficient) mice, as well as AS mice infected with sh-NC or sh-USP7 adenovirus plasmids for 24 h. (b) H&E staining showed the aortic morphology in normal mice and AS (ApoE-deficient) mice, as well as AS mice infected with sh-NC or sh-USP7 adenovirus plasmids for 24 h. Scale bar, 100 μm. ***p < 0.001, AS vs normal, ###*p* < 0.001, AS + sh-USP7 vs AS + sh-NC. NC, negative control; AS, atherosclerosis.

Immunoblot assays revealed high expression of USP7 in the aortic tissues of ApoE-deficient mice (AS mice) (Figure 1a). The silencing efficiency of USP7 shRNA adenovirus plasmids was confirmed in the aortic tissues of AS mice (Figure 1a). H&E staining showed that the aortic intima of normal mice was thin and without lumen stenosis. In contrast, AS mice displayed significant atherosclerotic lesions, characterized by thickening of the intima and the formation of atherosclerotic plaques (Figure 1b). However, USP7 depletion significantly improved the morphological characteristics of AS in these mice compared to those infected with sh-NC adenovirus (Figure 1b). Specifically, the intima-media thickness in USP7 knockdown mice was reduced by 42% compared to control AS mice (*p* < 0.001). Therefore, ablation of USP7 improved the AS morphological characteristics in AS mice.

### 3.2 Knockdown of USP7 suppressed inflammation in AS mice

**Figure 2:**
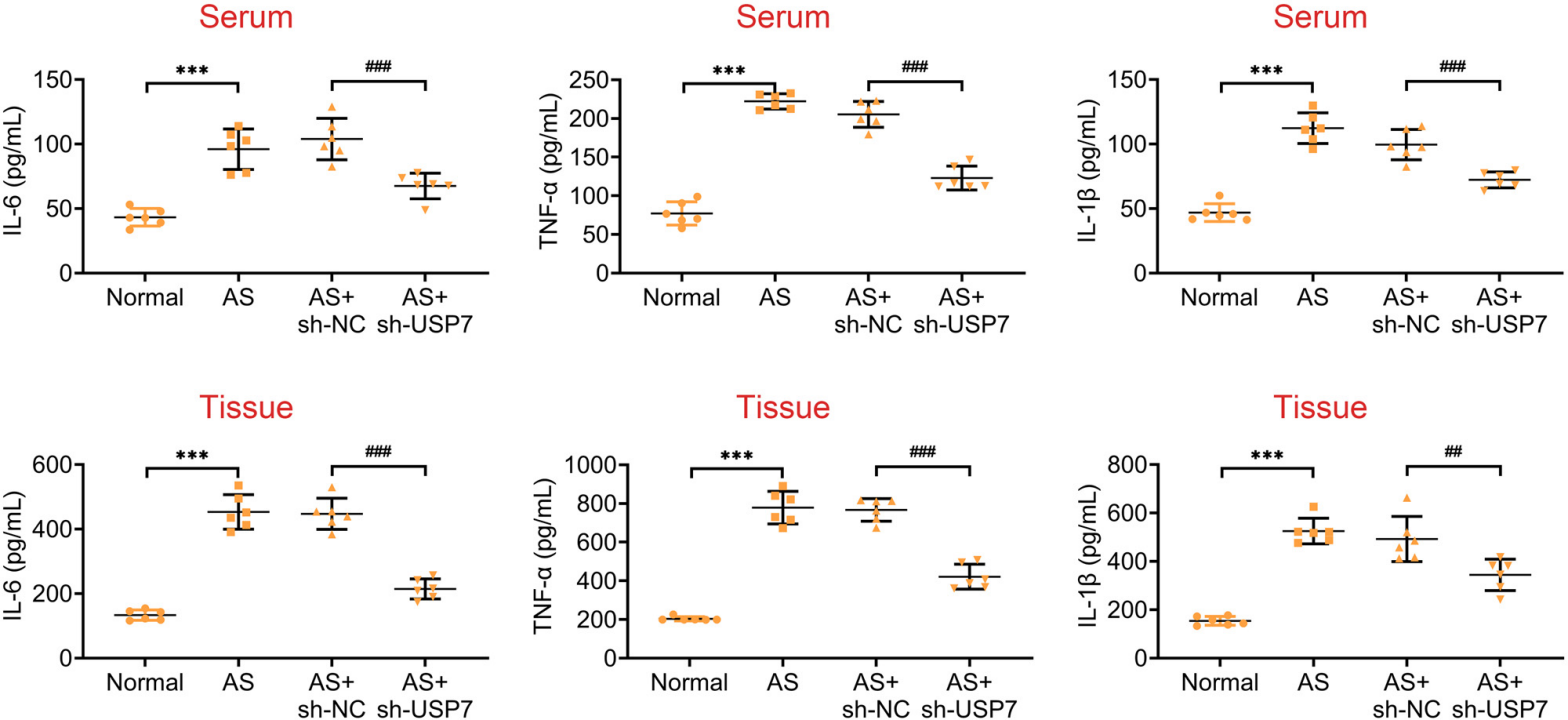
ELISA showed the secretion of IL-6, TNF-α, and IL-1β in the serum (up) and aortic tissues (down) of normal mice and AS (ApoE-deficient) mice, as well as AS mice infected with sh-NC or sh-USP7 adenovirus plasmids for 24 h.

ELISA was used to measure the levels of inflammatory factors, including IL-6, TNF-α, and IL-1β, in the serum and aortic tissues of normal and AS mice following USP7 depletion. We observed that the secretion of these factors was increased in both the serum and aortic tissues of AS mice compared to normal mice (Figure 2). However, USP7 depletion significantly suppressed the secretion of these factors in both the serum and aortic tissues of AS mice, indicating reduced inflammation (Figure 2). Specifically, the levels of IL-6, TNF-α, and IL-1β were decreased by 35, 40, and 38%, respectively, in USP7 knockdown mice (*p* < 0.01). Therefore, ablation of USP7 effectively suppressed inflammation in AS mice.

### 3.3 Depletion of USP7 restrained oxidative stress in AS mice

**Figure 3:**
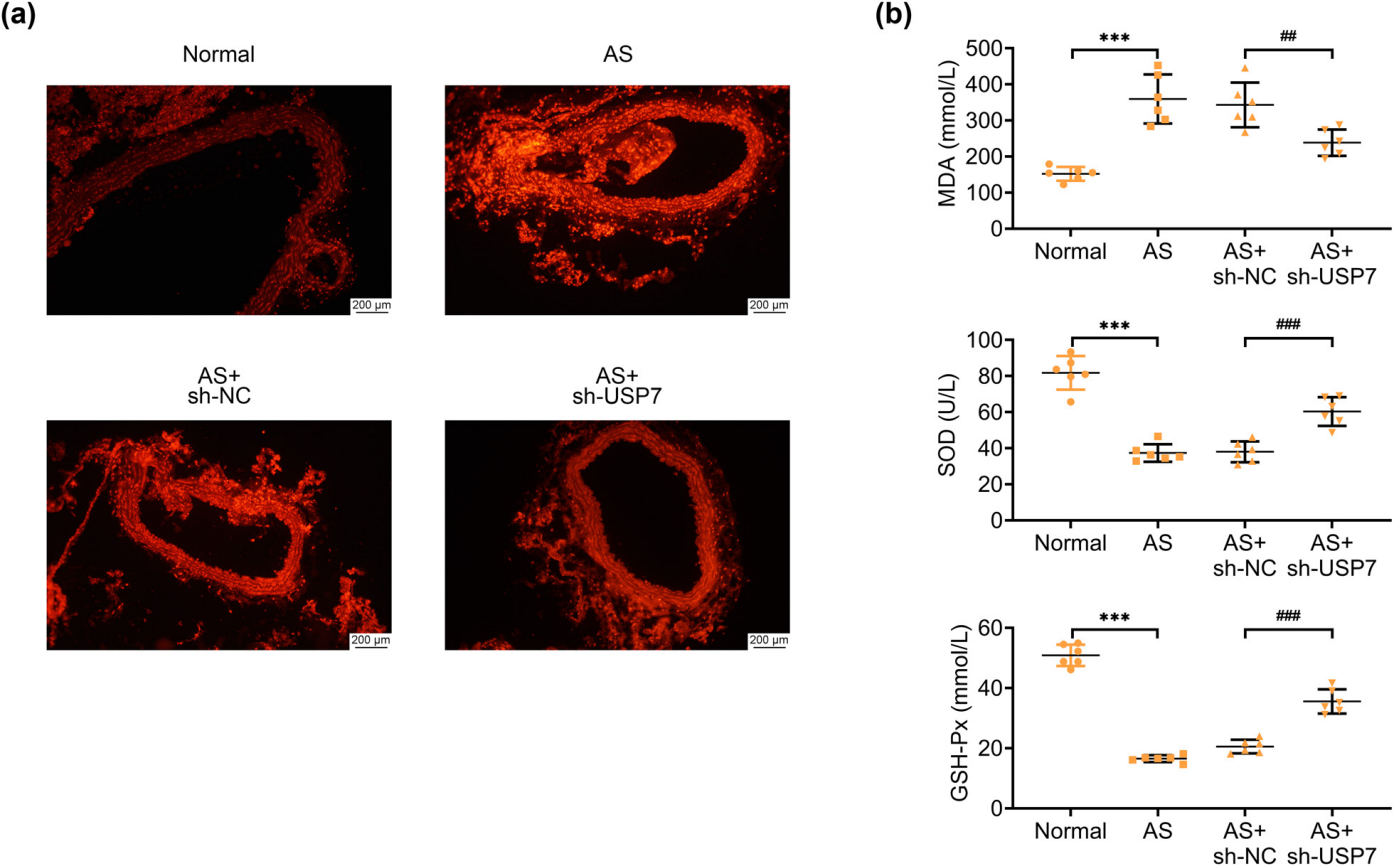
Depletion of USP7 restrained oxidative stress in AS mice. (a) DHE staining showed the oxidative stress (ROS levels) in the aortic tissues of normal mice and AS (ApoE-deficient) mice, as well as AS mice infected with sh-NC or sh-USP7 adenovirus plasmids for 24 h. Scale bar, 200 μm. (b) Levels of MDA, SOD, and GSH-Px in the aortic tissues of normal mice and AS (ApoE-deficient) mice, as well as AS mice infected with sh-NC or sh-USP7 adenovirus plasmids for 24 h, were measured using corresponding kits. ***p < 0.001, AS vs normal, ##*p* < 0.01, ###*p* < 0.001, AS + sh-USP7 vs AS + sh-NC. NC, negative control; AS, atherosclerosis.

DHE staining showed that the staining intensity of DHE was significantly increased in the aortic tissues of AS mice compared to normal mice, indicating elevated ROS production and oxidative stress (Figure 3a). Interestingly, USP7 ablation reduced the DHE staining intensity in the aortic tissues of AS mice, suggesting suppression of oxidative stress (Figure 3a). Consistent with this observation, we measured the levels of MDA, SOD, and GSH-Px. We found increased MDA levels and decreased SOD and GSH-Px levels in the aortic tissues of AS mice compared to normal mice (Figure 3b). However, USP7 ablation reversed these alterations, indicating a reduction in oxidative stress (Figure 3b). Therefore, depletion of USP7 effectively restrained oxidative stress in AS mice.

### 3.4 USP7 knockdown blocked the lipid accumulation of aortic tissues in AS mice

**Figure 4:**
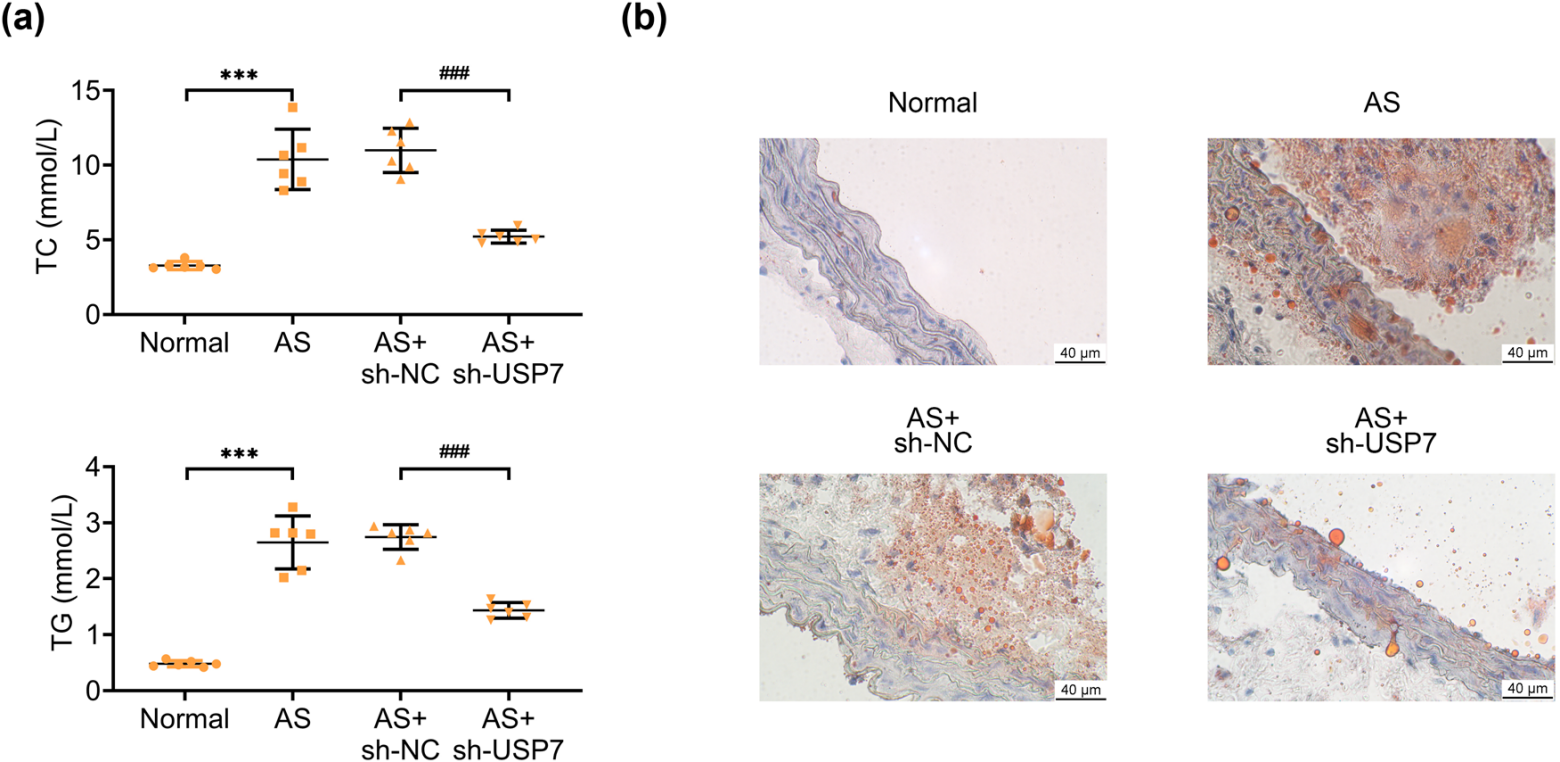
USP7 knockdown blocked the lipid accumulation of aortic tissues in AS mice. (a) TC and TG levels in the aortic tissues of normal mice and AS (ApoE-deficient) mice, as well as AS mice infected with sh-NC or sh-USP7 adenovirus plasmids for 24 h, were detected using corresponding kits. (b) Oil Red O staining showed the degree of lipid accumulation in the aortic tissues of normal mice and AS (ApoE-deficient) mice, as well as AS mice infected with sh-NC or sh-USP7 adenovirus plasmids for 24 h. Scale bar, 40 μm. ***p < 0.001, AS vs normal, ###*p* < 0.001, AS + sh-USP7 vs AS + sh-NC. NC, negative control; AS, atherosclerosis.

Next, we examined the effects of USP7 depletion on lipid accumulation in the aortic tissues of AS mice. Elevated levels of TG and TC were observed in the aortic tissues of AS mice compared to normal mice, indicating lipid accumulation (Figure 4a). However, USP7 depletion significantly reduced the levels of these lipids in the aortic tissues of AS mice (Figure 4a). Similarly, Oil Red O staining revealed lipid accumulation in the aortic tissues of AS mice (Figure 4b). USP7 depletion markedly suppressed this lipid accumulation (Figure 4b). Specifically, TC and TG levels in aortic tissues were decreased by 33 and 37%, respectively (*p* < 0.001). Therefore, USP7 knockdown effectively blocked lipid accumulation in the aortic tissues of AS mice.

### 3.5 USP7 knockdown inhibited EZH2 expression in AS mice

**Figure 5:**
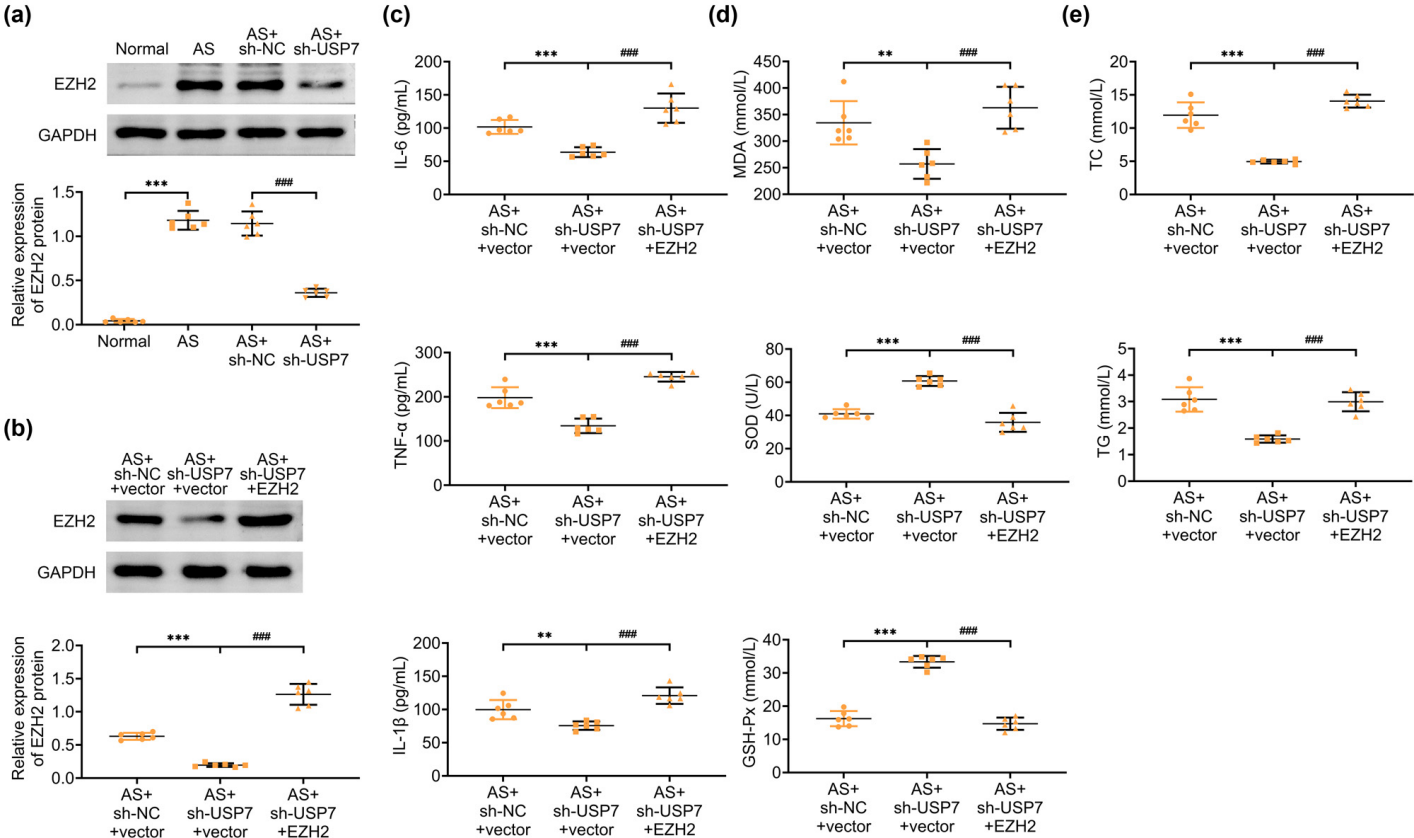
USP7 knockdown inhibited EZH2 expression in AS mice. (a) Immunoblot assays showed the expression of EZH2 in the aortic tissues of normal mice and AS (ApoE-deficient) mice, as well as AS mice infected with sh-NC or sh-USP7 adenovirus plasmids for 24 h. (b) Immunoblot assays showed the expression of EZH2 in the aortic tissues of AS mice infected with sh-NC or sh-USP7 and EZH2 adenovirus plasmids. (c) ELISA showed the secretion of IL-6, TNF-α, and IL-1β in the aortic tissues of AS mice infected with sh-NC or sh-USP7 and EZH2 adenovirus plasmids for 24 h. (d) Levels of MDA, SOD, and GSH-Px in the aortic tissues of AS mice infected with sh-NC or sh-USP7 and EZH2 adenovirus plasmids for 24 h were measured using corresponding kits. (e) TC and TG levels in the aortic tissues of AS mice infected with sh-NC or sh-USP7 and EZH2 adenovirus plasmids were detected using corresponding kits. **p < 0.01, ***p < 0.001, AS vs normal, ##*p* < 0.01, ###*p* < 0.001, AS + sh-USP7 vs AS + sh-NC. NC, negative control; AS, atherosclerosis.

Lastly, we investigated the potential underlying mechanism via which USP7 depletion might suppress AS progression. Immunoblot assays showed that EZH2 expression was increased in the aortic tissues of AS mice (Figure 5a). However, USP7 depletion led to a decrease in EZH2 expression (Figure 5a). Furthermore, EZH2 adenovirus infection further increased EZH2 expression in the aortic tissues of USP7-depleted AS mice (Figure 5b). Interestingly, ELISA results indicated that while USP7 depletion suppressed the secretion of inflammatory factors, EZH2 overexpression further increased their secretion in the aortic tissues of AS mice with USP7 knockdown (Figure 5c). Similarly, EZH2 overexpression increased MDA levels and decreased SOD and GSH-Px levels in the aortic tissues of AS mice with USP7 knockdown (Figure 5d). Additionally, the reduced levels of TC and TG due to USP7 knockdown were reversed by EZH2 overexpression in the aortic tissues of AS mice (Figure 5e). Collectively, these findings suggest that USP7 knockdown inhibits EZH2 expression in AS mice.

## 4 Discussion

AS is a major cardiovascular disease affecting various populations worldwide. Its epidemiological characteristics indicate a close relationship with age, gender, genetic factors, and lifestyle factors such as diet, exercise, and smoking [16,17]. Present treatment strategies primarily focus on controlling blood pressure, lowering blood lipids, improving endothelial function, and anti-inflammatory measures. The formation of AS has been found to be closely linked to lipid accumulation, inflammatory response, and oxidative stress. The accumulation of LDL in the vascular wall is a key factor, leading to impaired endothelial cell (EC) function, recruitment and activation of inflammatory cells, and increased oxidative stress. These processes interact, accelerating the formation and development of atherosclerotic plaques [16]. Therefore, comprehensive interventions, including lifestyle changes and pharmacological treatments, are crucial for the prevention and management of AS. Our data confirmed that USP7 knockdown suppressed AS progression in ApoE-deficient mice. Furthermore, USP7 depletion improved lipid accumulation, suppressed oxidative stress, and reduced inflammation in AS mice, which are consistent with previous studies on the role of USP7 in lipid metabolism and inflammation. For instance, Ni et al. demonstrated that USP7 mediates pathological hepatic *de novo* lipogenesis by promoting the stabilization and transcription of ZNF638.

USP7 is a protease that primarily regulates protein stability and function through deubiquitination. It plays an essential role in cell cycle regulation, DNA damage repair, immune response, and tumor suppression by modulating various proteins such as the tumor suppressor p53, its E3 ubiquitin ligase MDM2, and other signaling molecules [18]. Although research on the relationship between USP7 and blood vessels is relatively limited, some studies suggest that USP7 may influence EC function and vascular stability. For instance, USP7 has been found to regulate key proteins in ECs, affecting their proliferation, migration, and tubulogenesis, which are essential for angiogenesis and vascular repair [19]. Additionally, USP7 may indirectly impact vascular health through its effects on inflammation and immune responses [20]. However, the specific role and mechanisms of USP7 in vascular biology require further investigation. Our experiments using ApoE-deficient mice confirmed that USP7 affects AS progression by mediating lipid accumulation, oxidative stress, and inflammation. Our data suggest that USP7 plays a key role in AS progression. Specifically, USP7 may regulate AS progression through its interaction with key signaling pathways involved in lipid metabolism and inflammatory responses. Additionally, USP7 might influence EZH2 stability and activity, further modulating gene expression profiles associated with atherosclerotic plaque formation and progression. Therefore, our findings highlight the critical role of USP7 in AS progression and suggest it as a potential therapeutic target.

EZH2 is a histone methyltransferase that regulates gene expression primarily by catalyzing the trimethylation of H3K27me3. As the catalytic subunit of the PRC2, EZH2 is involved in various biological processes, including cell differentiation, proliferation, and tumorigenesis. Recent studies have shown that EZH2 plays a significant role in the development of AS. Elevated expression of EZH2 has been observed in vascular smooth muscle cells (VSMCs) and ECs associated with AS [6]. In VSMCs, overexpression of EZH2 promotes cell proliferation and migration, which are key processes in the formation and progression of atherosclerotic plaques [21]. Additionally, EZH2 can influence the progression of AS by regulating the expression of genes related to inflammation and immune responses. Although the precise mechanisms of EZH2 in AS are not fully understood, these findings suggest that EZH2 could be an important regulator in the pathogenesis of AS and a potential target for future therapeutic interventions. Previous studies have indicated that the interaction between USP7 and EZH2 can also play a role in the regulation of immune response and inflammation, processes that are relevant to conditions such as autoimmunity and cancer. Importantly, our data confirmed that USP7 knockdown inhibited EZH2 expression and consequently suppressed AS progression. However, the precise mechanisms underlying this regulation need further investigation.

This study had some limitations that should be acknowledged. For instance, the use of a single animal model (ApoE-deficient mice) may not fully capture the complexity of AS in humans. Further validation of our findings in human tissues and additional animal models is necessary to confirm the generalizability of our results. Additionally, exploring the long-term effects of USP7 inhibition and its potential side effects could be crucial for translating these findings into clinical applications.

Future studies could utilize additional animal models, such as LDL receptor-deficient mice, to further validate the role of USP7 in AS. In addition, investigating the effects of USP7 inhibition in human vascular tissues and atherosclerotic plaques could be essential in providing more insights into this topic. Moreover, detailed mechanistic studies are needed to identify the downstream targets of EZH2 and the specific pathways involved in USP7-mediated regulation of lipid metabolism and inflammation. These studies could provide deeper insights into the molecular mechanisms at play. Lastly, evaluating the therapeutic potential and safety of USP7 inhibitors in preclinical and clinical settings will be crucial for translating these findings into viable treatments for AS.

In conclusion, this study revealed that USP7 ablation could alleviate AS in ApoE-deficient mice by regulating EZH2, thereby demonstrating potential as a promising therapeutic target for AS.
